# Supplementary material for: Spontaneous spin-valley polarization in NbSe2 at a van der Waals interface
Source: Nat Commun. 2022 Sep 15;13:5129. doi: 10.1038/s41467-022-32810-2 (PMC9477796; doi:10.1038/s41467-022-32810-2)
Supplement: Supplementary file 1 — Supplementary Information [file 41467_2022_32810_MOESM1_ESM.pdf]

Supplementary Information for

Spontaneous spin-valley polarization in $NbSe_2$ at a van der Waals interface

Hideki Matsuoka[1,2], Tetsuro Habe[3,4], Yoshihiro Iwasa[1,2], Mikito Koshino[5], and Masaki Nakano[1,2,*]

[1]*Quantum-Phase Electronics Center and Department of Applied Physics, the University of Tokyo, Tokyo 113-8656, Japan.*

[2]*RIKEN Center for Emergent Matter Science (CEMS), Wako 351-0198, Japan.*

[3]*Department of Applied Physics, Hokkaido University, Hokkaido 060-0808, Japan.*

[4]*Nagamori Institute of Actuators, Kyoto University of Advanced Science, Kyoto 615-0096, Japan.*

[5]*Department of Physics, Osaka University, Osaka 560-0043, Japan.*

[*]e-mail: nakano@ap.t.u-tokyo.ac.jp

Supplementary Notes:

1. Sample fabrication and characterization.
2. The *R*-*T* curves of the representative samples.
3. The detailed AHE data of the representative samples.
4. The angle dependence of the AHE of another sample.
5. The angle dependence of the normal Hall effect.
6. Other calculation results on monolayer $NbSe_2$.
7. Calculation results on bilayer $NbSe_2$.

1. Sample fabrication and characterization.

All the samples were fabricated by MBE by following our growth process[1-3]. The layer number was precisely designed by monitoring the RHEED intensity oscillations during the growth. Supplementary Figures 1a and 1b show typical RHEED intensity oscillations recorded during the growth of the initial V_5Se_8 layers and the following $NbSe_2$ layers, respectively. The actual layer numbers were confirmed by XRD measurements. Supplementary Figure 1c shows the out-of-plane XRD pattern of the $N = 6$ L sample used in this study. The strong diffraction peak was observed at around 14-15° with clear Laue oscillation, indicating high crystalline coherence along the out-of-plane direction. The complicated pattern could be well fitted by simulation calculated using the parameters of the layer number and the lattice constant for the respective layers, verifying the formation of the abrupt interface, which could be in fact confirmed by STEM measurements as shown in Supplementary Fig. 1d.

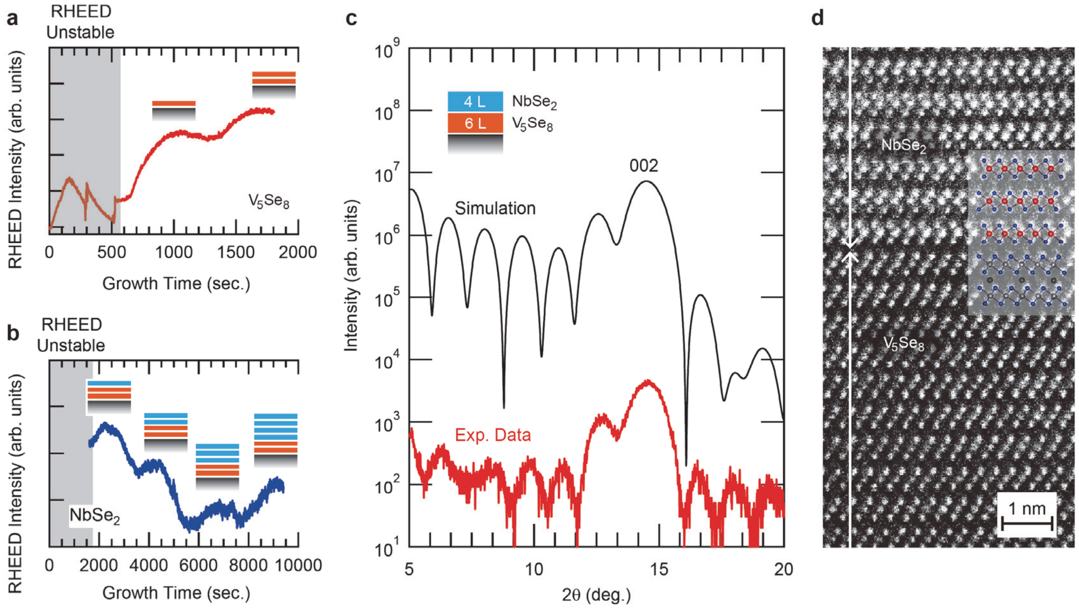

Supplementary Figure 1 | Structural characterization of the samples.
a,b, The RHEED intensity oscillations recorded during the growth of (**a**) the initial V_5Se_8 layers and (**b**) the following $NbSe_2$ layers. **c**, The out-of-plane XRD pattern of the $N = 6$ L sample used in this study together with the simulated pattern. **d**, The overall STEM image of a typical sample.

2. The *R-T* curves of the representative samples.

As we wrote in the main text, we consider that the electrical conductions of the $N < 3$ L samples in the low temperature regime are governed by the 4 L-thick $NbSe_2$ layer. Supplementary Figure 2 shows the normalized *R-T* curves of the individual films and that of the Nb/V heterostructure sample with $N = 2.0$ L. The V_5Se_8 individual film showed metallic behavior in the thick-enough regime (30 L), whereas it exhibited weakly insulating behavior in the thin limit (3 L) as we reported in the previous study[1]. On the other hand, the $NbSe_2$ individual film exhibited metallic behavior down to the thin limit (3 L)[2], although small upturn was still visible in the low temperature regime. The Nb/V heterostructure sample with $N = 2.0$ L exhibited metallic behavior down to the lowest temperature, whose electrical conduction should be dominated by $NbSe_2$. The characteristic kink-like behavior observed in the heterostructure sample corresponds to the ferromagnetic transition temperature[3].

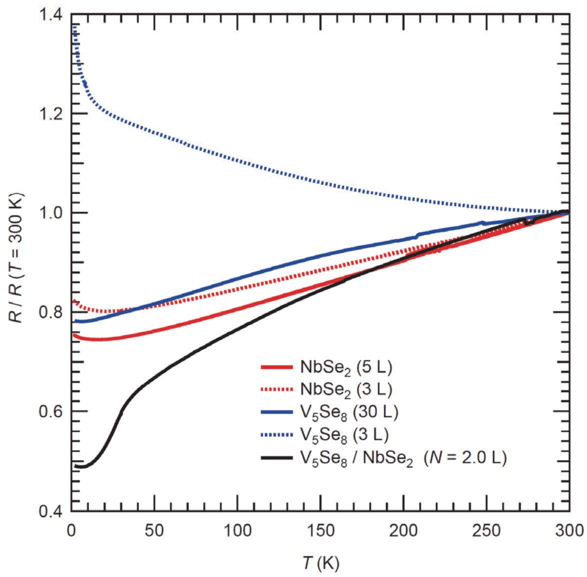

Supplementary Figure 2 | The *R-T* curves of the representative samples.
The normalized *R-T* curves of the 30 L and 3 L-thick V_5Se_8 individual films, the 5 L and 3 L-thick $NbSe_2$ individual films, and the Nb/V heterostructure with $N = 2.0$ L.

3. The detailed AHE data of the representative samples.

Supplementary Figures 3a-d show the anti-symmetrized AHE data of the representative samples taken at various temperatures. The data at $T = 2$ K for all four samples are the same as those shown in Fig. 2a. The temperature dependence of the R_{AH} at the saturated regime, $R_{AH, sat}$, of those samples are plotted in Fig. 2b. The normal Hall components were subtracted from all the data. The sign of the AHE did not change for the $N = 6$ L sample, while it was inverted from negative to positive below $T \sim$ 10-20 K for the $N < 3$ L samples.

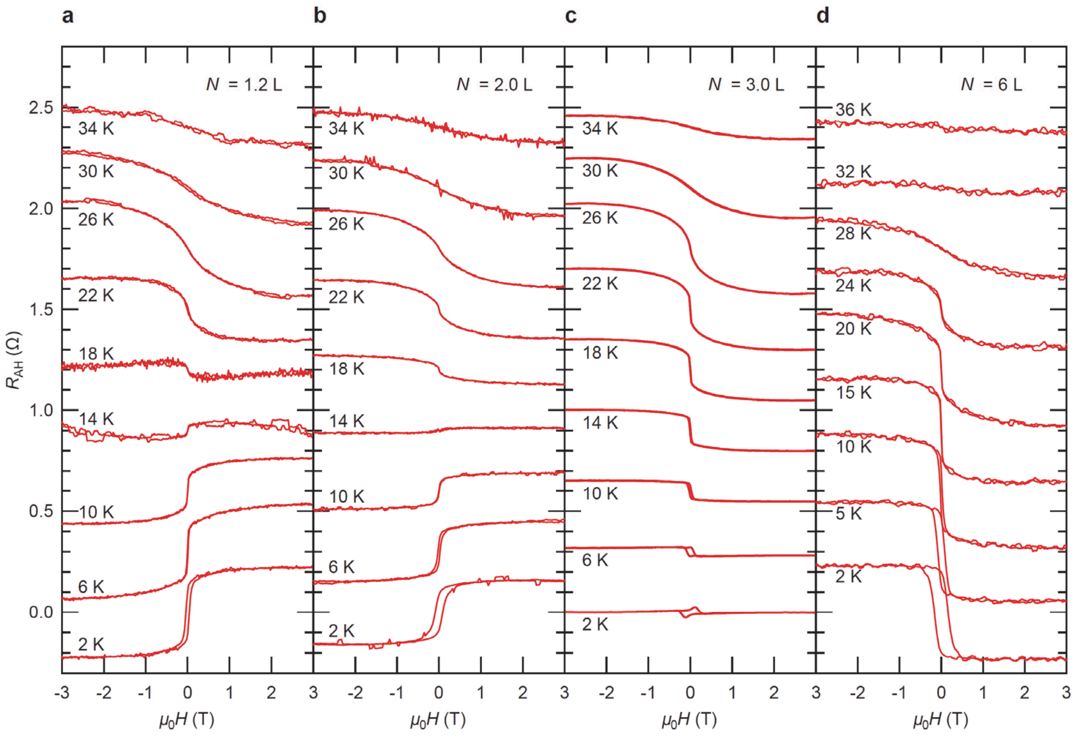

Supplementary Figure 3 | The detailed AHE data of the representative samples.
a-d, The detailed AHE data of the $N =$ (**a**) 1.2 L, (**b**) 2.0 L, (**c**) 3.0 L, and (**d**) 6 L samples, respectively, taken at various temperatures. The data at $T = 2$ K are the same as those shown in Fig. 2a. The normal Hall components were subtracted from all the data.

4. The angle dependence of the AHE of another sample.

Supplementary Figure 4a displays the AHE of the N = 1.2 L sample at T = 2 K with different field angles (θ), and Supplementary Fig. 4b shows the normalized AHE signal as a function of θ taken at $\mu_0 H$ = 9 T. The data well reproduced those for the N = 2.0 L sample shown in Figs. 3a and 3c.

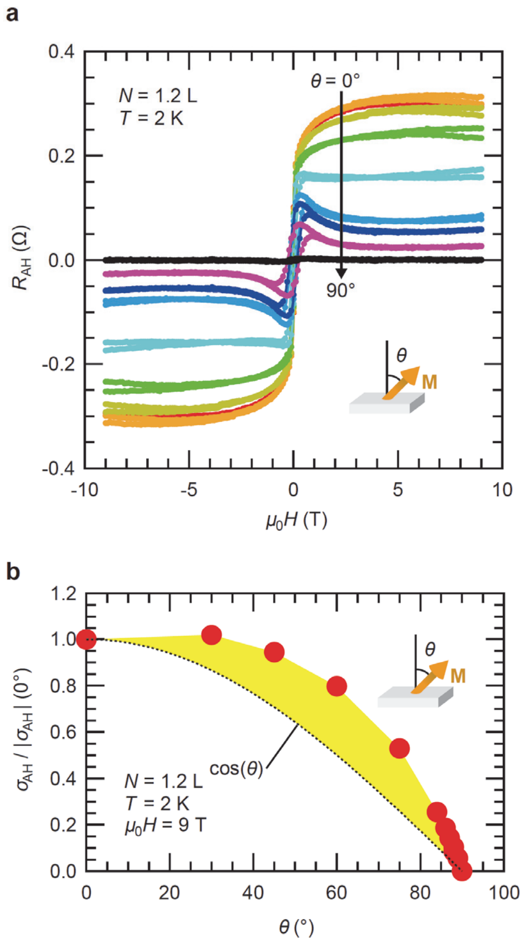

Supplementary Figure 4 | The angle dependence of the AHE of another sample.
a, The R_{AH} of the N = 1.2 L sample at T = 2 K with different θ. The configuration of the field angle θ is shown in the inset. **b,** The σ_{AH} at $\mu_0 H$ = 9 T plotted against θ. The data is normalized by the σ_{AH} at $\theta = 0^{\circ}$. The black dotted line is cos (θ) relative to the σ_{AH} at $\theta = 0^{\circ}$, and the yellow region highlights a deviation from cos (θ).

5. The angle dependence of the normal Hall effect.

We verified the field angles (θ) by checking the angle dependence of the normal Hall components. Supplementary Figures 5a and 5b show the angle dependence of the Hall coefficient (R_H) for (**a**) the N = 1.2 L sample and (**b**) the N = 2.0 L sample, respectively, deduced from the linear fittings of the Hall-effect data at the high-enough field regimes. In contrast to the AH components shown by the red symbols (which are the same as those shown in Supplementary Fig. 4b for N = 1.2 L and in Fig. 3c for N = 2.0 L), the normal Hall components simply follow cos (θ) for both samples, excluding a possibility of the sample misalignment to be the origin of the deviation of the AH component from cos (θ).

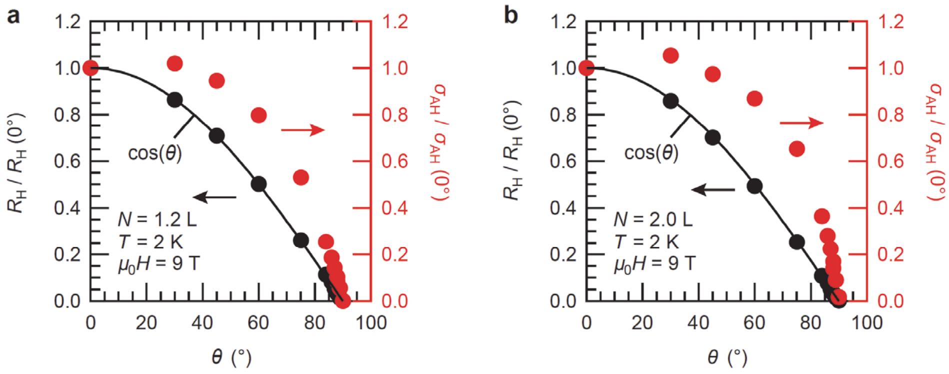

Supplementary Figure 5 | The angle dependence of the normal Hall effect.
The angle dependence of the normal Hall components (black symbols) and the AH components (red symbols) of the N = (**a**) 1.2 L and (**b**) 2.0 L samples, respectively, taken at T = 2 K and $\mu_0 H$ = 9 T. The data are normalized by the values at θ = 0º. The black solid lines are cos (θ) relative to the value at θ = 0º.

6. Other calculation results on monolayer $NbSe_2$.

In the main text, we discuss the calculation results with the fixed exchange field |**M**| = 40 meV, but in reality the exchange field is unknown. In this section, we provide the calculation results with different exchange fields, as well as the results on the angle dependence of the AHE at different energies.

(I) The angle dependence of the AHE with different exchange fields.

Supplementary Figure 6a shows the angle dependence of the σ_{xy} with different |**M**| at $E = E_F$, and Supplementary Fig. 6b shows the magnitude of a deviation from cos (θ) at $\theta = 20^o$ as a function of |**M**|. The largest deviation is achieved when |**M**| = 40 meV, corresponding to the situation that the corner of the FS of monolayer $NbSe_2$ near the Γ valley is contacted with the peak of the emergent Berry curvature surrounding the K valleys originating from the spin-degenerate nodal lines as we discussed in the main text (see Fig. 5f). We however note that such a deviation from cos (θ) could be observed in a rather broad range of the exchange field from a few millielectronvolt to a hundred millielectronvolt (see Supplementary Fig. 6b).

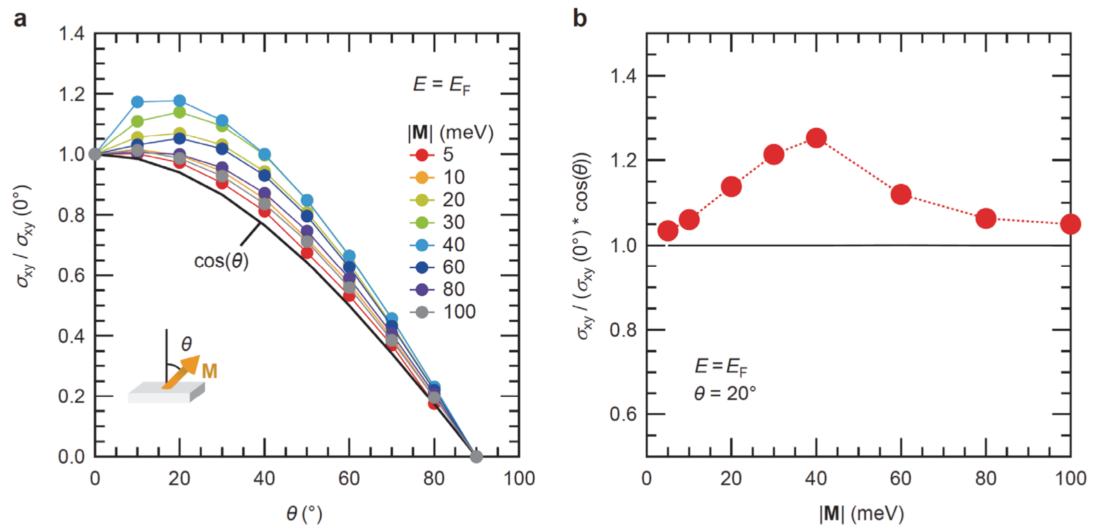

Supplementary Figure 6 | The angle dependence of the AHE with different exchange fields.

a, The angle dependence of the σ_{xy} with different |**M**| at $E = E_F$ calculated from the band structure of monolayer $NbSe_2$. The configuration of θ is shown in the inset. **b,** The magnitude of a deviation of the σ_{xy} from cos (θ) at $\theta = 20^o$ as a function of |**M**|.

(II) The angle dependence of the AHE at different energies.

Supplementary Figure 7a shows the energy dependence of the σ_{xy} with $|\mathbf{M}|$ = 40 meV for different θ calculated from the band structure of monolayer $NbSe_2$ shown in Fig. 4d, and Supplementary Fig. 7b shows the corresponding angle dependence of the σ_{xy} at two representative energies. A deviation of the σ_{xy} from cos (θ) could be observed when $E = E_F$ (red symbols in **b**) but not observed when $E = E_F$ + 580 meV (blue symbols in **b**). This suggests that the observed phenomena associated with the emergence of the Berry curvature with the in-plane magnetization are unique to group-V metallic *H*-type TMDCs such as $NbSe_2$ and TaS_2 but missing in group-VI semiconducting *H*-type TMDCs such as MoS_2 and WSe_2, and that the crossing of the FS and the nodal lines near the Γ valley plays a key role for the enhancement of the AHE signal with the in-plane fields.

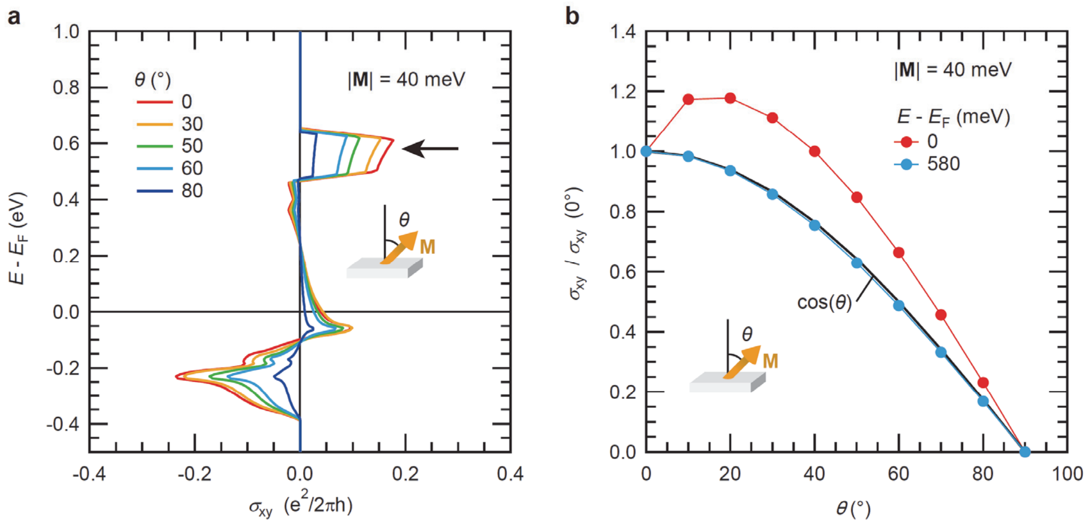

Supplementary Figure 7 | The angle dependence of the AHE at different energies. **a,** The σ_{xy} as a function of energy with $|\mathbf{M}|$ = 40 meV for different θ calculated from the band structure of monolayer $NbSe_2$. The configuration of θ is shown in the inset. **b,** The angle dependence of the σ_{xy} with $|\mathbf{M}|$ = 40 meV at $E = E_F$ (red symbols) and at $E = E_F$ + 580 meV (blue symbols), corresponding to the energy indicated by an arrow in **a**.

7. Calculation results on bilayer $NbSe_2$.

In the main text, we discuss the band structure of monolayer $NbSe_2$, but in reality our samples have multilayer $NbSe_2$. In this section, we discuss the band structure of bilayer $NbSe_2$ under the exchange field, and demonstrate that a physical picture based on monolayer $NbSe_2$ proposed in the main text could be applicable to bilayer $NbSe_2$ as well. Multilayer $NbSe_2$ should provide essentially the same results as those of bilayer $NbSe_2$ as long as a proximity effect is limited to one layer in contact with a ferromagnet.

(I) The band structure of bilayer $NbSe_2$ without the exchange field.

Supplementary Figure 8a shows a schematic crystal structure of bilayer $NbSe_2$. As compared to monolayer $NbSe_2$ with broken in-plane inversion symmetry, bilayer $NbSe_2$ has an inversion center due to the $2H_a$ stacking, where two monolayers are stacked with 180° in-plane rotation from each other. Consequently, if we ignore the interlayer interaction, the corresponding band structure could be considered as the overlay of two monolayer bands with 180° in-plane rotation, where the up-spin/down-spin bands of the first layer (1, ↑)/(1, ↓) are fully degenerate to the down-spin/up-spin bands of the second layer (2, ↓)/(2, ↑). Then, when we consider the interlayer interaction, the bands of the different layers with the same spins get hybridized [*i.e.*, (1, ↑) hybridizes with (2, ↑), and (1, ↓) hybridizes with (2, ↓)]. The effect of this hybridization is maximum at the Γ point where the out-of-plane d_{z^2} orbitals have the largest contribution[4,5], resulting in the significant modification of the bands near the Γ valley from those of monolayer $NbSe_2$.

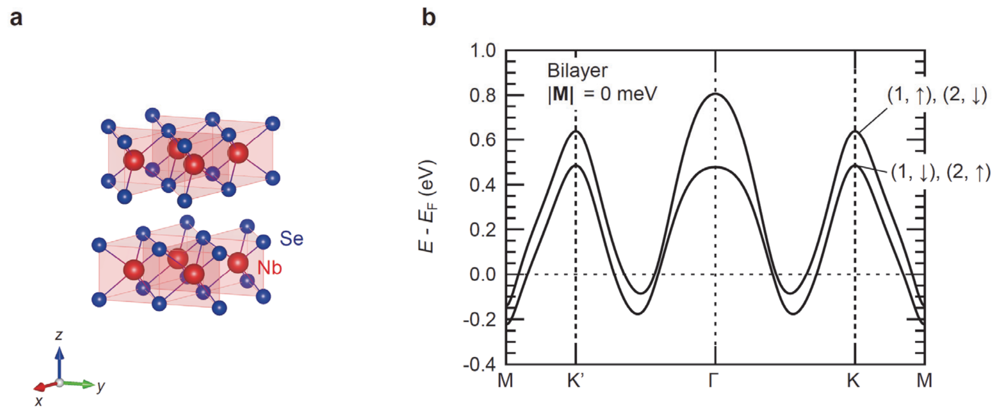

Supplementary Figure 8 | The band structure of bilayer $NbSe_2$ without the exchange field.

a, A schematic crystal structure of bilayer $NbSe_2$ with the $2H_a$ stacking. **b,** The band structure of bilayer $NbSe_2$. The up-spin/down-spin bands of the first layer are fully degenerate to the down-spin/up-spin bands of the second layer.

On the other hand, the effect is minimum at the K and K' points where the in-plane d_{xy} and $d_{x^2-y^2}$ orbitals have the largest contribution[4,5], keeping the characters of the bands near the K and K' valleys to be similar to those before hybridization. Moreover, this interlayer interaction opens an energy gap along the Γ-M line, where all the four bands are originally degenerate before hybridization. The resultant band structure is shown in Supplementary Fig. 8b. A finite energy gap is visible between the upper and lower bands, both of which have two-fold degeneracy.

(II) The band structure of bilayer $NbSe_2$ under the exchange field.

Now, let us consider the situation when the first layer of bilayer $NbSe_2$ is subjected to the out-of-plane exchange field. Supplementary Figure 9a shows the band structure of bilayer $NbSe_2$ with the exchange field (|**M**| = 40 meV) applied parallel to the *c*-axis, where the bands of the first layer (1, ↑)/(1, ↓) are shifted down/up due to Zeeman effect as is the case for monolayer $NbSe_2$ with the exchange field (see Fig. 4d), while the bands of the second layer (2, ↓)/(2, ↑) remain unchanged, resulting in the lifting of the degeneracy between (1, ↑)/(1, ↓) and (2, ↓)/(2, ↑). Supplementary Figure 9b shows the magnified view of the band structure around the dotted rectangular region in Supplementary Fig. 9a, where the non-degenerate four bands could be clearly recognized. Interestingly, those densely-distributed bands accompany very large Berry curvature as representatively shown for one specific band highlighted by red color on the bottom of Supplementary Fig. 9b. Such large Berry curvature in this narrow-gap region should be originating from hybridization of the orbital pseudospins by the interlayer interaction, where d_{z^2} and

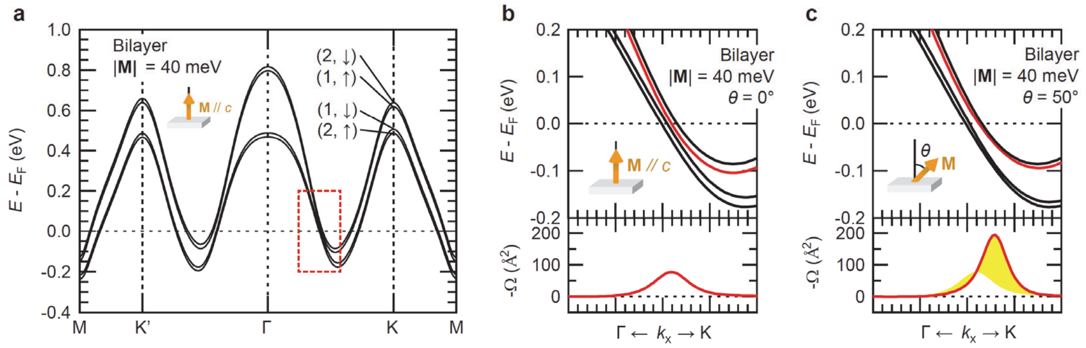

Supplementary Figure 9 | The band structure of bilayer $NbSe_2$ under the exchange field.

a, The band structure of bilayer $NbSe_2$ with the exchange field (|**M**| = 40 meV) applied parallel to the *c*-axis (**M**//*c*). **b,c,** The magnified views of the band structures and the Berry curvature in the dotted rectangular region in **a** for (**b**) $\theta = 0^\circ$ and (**c**) $\theta = 50^\circ$. The absolute value of the exchange field is fixed to |**M**| = 40 meV.

$d_{x^2-y^2} \pm id_{xy}$ are associated with the up- and down-pseudospin, respectively[5-7]. We note that the real spins do not contribute to the Berry curvature in this case, as the up-spin band and the down-spin band are not hybridized by the out-of-plane exchange field.

Those band structure and the Berry curvature are largely modulated when the exchange field is tilted to the in-plane direction as shown in Supplementary Fig. 9c. We observe the emergence of the additional Berry curvature as is the case for monolayer $NbSe_2$, part of which should be originating from hybridization of the up-spin band and the down-spin band by the in-plane magnetization. However, there should be another contribution from the orbital pseudospins, which should be also mixed by the in-plane magnetization and generate additional Berry curvature. As a result, a deviation of the σ_{xy} from cos (θ) becomes much more pronounced in bilayer $NbSe_2$ as will be shown in the next section.

(III) The angle dependence of the AHE of bilayer $NbSe_2$.

Supplementary Figure 10a shows the energy dependence of the σ_{xy} for different θ calculated from the band structure of bilayer $NbSe_2$ shown in Supplementary Fig. 9a. The sign of σ_{xy} is positive at $E = E_F$ as is the case for monolayer $NbSe_2$, which is consistent to the experimental results. Supplementary Figure 10b shows the angle dependences of the σ_{xy} at $E = E_F$ for monolayer and bilayer $NbSe_2$. Clear deviations from cos (θ) are visible for both cases, suggesting that a similar mechanism associated with the emergence

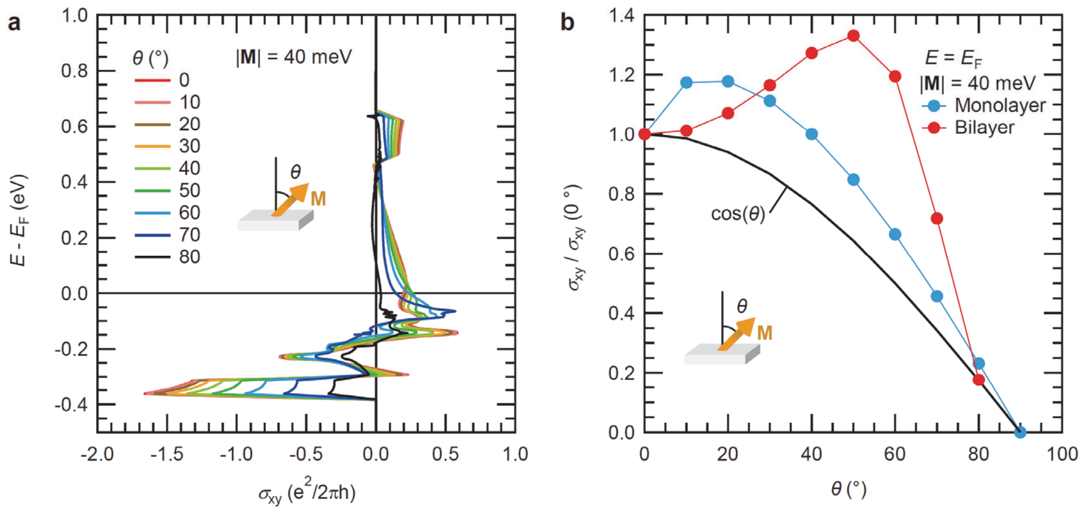

Supplementary Figure 10 | The angle dependence of the AHE of bilayer $NbSe_2$.
a, The σ_{xy} as a function of energy with $|\mathbf{M}| = 40$ meV for different θ calculated from the band structure of bilayer $NbSe_2$. The configuration of θ is shown in the inset. **b,** The angle dependence of the σ_{xy} with $|\mathbf{M}| = 40$ meV at $E = E_F$ for monolayer $NbSe_2$ (blue symbols) and bilayer $NbSe_2$ (red symbols).

of the additional Berry curvature with the in-plane magnetization should be at work for both monolayer and bilayer cases. The details of the behavior are however different, most likely because bilayer $NbSe_2$ has two origins for the additional Berry curvature, the real spins and the orbital pseudospins.

Supplementary References

1. Nakano, M. *et al.* Intrinsic 2D ferromagnetism in V_5Se_8 epitaxial thin films. *Nano Lett.* **19**, 8806-8810 (2019).
2. Matsuoka, H. *et al.* Angle dependence of H_{c2} with a crossover between the orbital and paramagnetic limits. *Phys. Rev. Research* **2**, 012064(R) (2020).
3. Matsuoka, H. *et al.* Spin-orbit-induced Ising ferromagnetism at a van der Waals interface. *Nano Lett.* **21**, 1807-1814 (2021).
4. Zhu, Z. Y., Cheng, Y. C. & Schwingenschlögl, U. Giant spin-orbit-induced spin splitting in two-dimensional transition-metal dichalcogenide semiconductors. *Phys. Rev. B* **84**, 153402 (2011).
5. Liu, G.-B., Shan, W.-Y., Yao, Y., Yao, W. & Xiao, D. Three-band tight-binding model for monolayers of group-VIB transition metal dichalcogenides. *Phys. Rev. B* **88**, 085433 (2013).
6. Xiao, D., Liu, G. Bin, Feng, W., Xu, X. & Yao, W. Coupled spin and valley physics in monolayers of MoS_2 and other group-VI dichalcogenides. *Phys. Rev. Lett.* **108**, 196802 (2012).
7. Xu, X., Yao, W., Xiao, D. & Heinz, T. F. Spin and pseudospins in layered transition metal dichalcogenides. *Nat. Phys.* **10**, 343-350 (2014).
